# Assessment of Silver Nanoparticles Derived from Brown Algae *Sargassum vulgare:* Insight into Antioxidants, Anticancer, Antibacterial and Hepatoprotective Effect

**DOI:** 10.3390/md22040154

**Published:** 2024-03-28

**Authors:** Ragaa A. Hamouda, Ebtehail S. Aljohani

**Affiliations:** 1 Department of Biology, College of Sciences and Arts Khulais, University of Jeddah, Jeddah 21959, Saudi Arabia; 2100257@uj.edu.sa; 2 Microbial Biotechnology Department, Genetic Engineering and Biotechnology Research Institute, University of Sadat City, Sadat City 32897, Egypt

**Keywords:** *Sargassum vulgare*, hepato-protective, anticancer, antibacterial, antioxidant

## Abstract

Algae are used as safe materials to fabricate novel nanoparticles to treat some diseases. Marine brown alga *Sargassum vulgare* are used to fabricate silver nanoparticles (Sv/Ag-NPs). The characterization of Sv/Ag-NPs was determined by TEM, EDX, Zeta potential, XRD, and UV spectroscopy. The Sv/Ag-NPs were investigated as antioxidant, anticancer, and antibacterial activities against Gram-positive bacteria *Bacillus mojavensis* PP400982, *Staphylococcus caprae* PP401704, *Staphylococcus capitis* PP402689, and *Staphylococcus epidermidis* PP403851. The activity of the Sv/Ag-NPs was evaluated as hepatoprotective in vitro in comparison with silymarin. The UV–visible spectrum of Sv/Ag-NPs appeared at 442 nm; the size of Sv/Ag-NPs is in range between 6.90 to 16.97 nm, and spherical in shape. Different concentrations of Sv/Ag-NPs possessed antioxidant, anticancer activities against (HepG-2), colon carcinoma (HCT-116), cervical carcinoma (HeLa), and prostate carcinoma (PC-3) with IC50 50.46, 45.84, 78.42, and 100.39 µg/mL, respectively. The Sv/Ag-NPs induced the cell viability of Hep G2 cells and hepatocytes treated with carbon tetrachloride. The Sv/Ag-NPs exhibited antibacterial activities against *Staphylococcus caprae* PP401704, *Staphylococcus capitis* PP402689, and *Staphylococcus epidermidis* PP403851. This study strongly suggests the silver nanoparticles derived from *Sargassum vulgare* showed potential hepato-protective effect against carbon tetrachloride-induced liver cells, and could be used as anticancer and antibacterial activities.

## 1. Introduction

Cancer has been categorized as a life-threatening problem in new medicine [1]. Approximately two million deaths globally are attributed to liver disease each year that is caused by cirrhosis complications, viral hepatitis, and hepatocellular cancer [2]. Numerous processes are carried out by the liver that maintain health, such as transforming nutrients into necessary molecules for the body, detoxifying harmful substances, and aiding in converting food into energy. Therefore, poor liver function can have an impact on the body [3]. Antimicrobial-resistant bacteria are an issue that is spreading quickly and could have disastrous effects [4]. Ninety percent of hospitalized patient infections in affluent nations are traced back to bacterial infections. Numerous bacterial diseases frequently have a catastrophic impact on the health of people living in poor nations. These people are more vulnerable to bacterial illnesses due to several reasons, including inadequate sanitation, parasite diseases, and malnourishment [5].

Antimicrobial agents inhibit and handle infections in humans and plants; these include antibiotics and antivirals [6]. Antimicrobial activity inhibits the growth of microbes, prevents their spread, and destroys microorganisms [7].

Nanotechnology is used to produce safe materials at the nano level to be inserted into the body of a living organism. Its applications in medicine include imaging, diagnosing diseases, and delivering medicines, thus helping doctors with treating many diseases. Due to the ability of nanotechnology to target specific cells and tissues, it is of great importance in the production of medicines, as linking nanoparticles to medicines increases efficiency [8]. Nanoparticles’ strongest antimicrobial effect against microorganisms is also having anticancer properties. Manufacturing nanoparticles using green biological techniques such as microorganisms, plants, and viruses is safer, more economical, and less toxic [9]. Green biosynthesized nanoparticles, which are particularly interesting, are produced by reducing metabolites from plant-derived products, and macro- and microorganisms. This is a superior technique to produce artificially physical or chemically generated nanoparticles that are inexpensive and less dangerous for human health and the environment [10]. Bacterial resistance can be overcome, in large part, by applying nanotechnology sensibly and effectively. Simultaneously, the combination of natural antimicrobials or alternative approaches with nanoparticles is being studied for its potential to treat multidrug-resistant bacteria [11].

There are many studies on using nanotechnology in liver fibrosis medication delivery. Combination therapy and targeted drug administration are made easier by improved internalization and penetration, made possible by nano-medicine [12]. A naturally occurring substance called silymarin is obtained from the *Silybum marianum* plant; this plant has the flavonoid taxifolin and at least seven other flavolignans. Due to the fact that silymarin can suppress free radicals formed by the metabolism of harmful drugs such ethanol, acetaminophen, and carbon tetrachloride, it has hepatoprotective and antioxidant properties [13]. The mechanisms of action of silymarin include various biochemical proceedings, such as the enhancement of the synthetic rate of ribosomal RNA (rRNA) species through the enhancement of polymerase I and rRNA transcription, caring after the cell membrane from radical-induced harm and the obstruction of the uptake of toxins such as α-amanitin [14].

Marine algae can be used in biogenic nanoparticles due to the eco-friendly, cost-effective, fast-acting, and energy-efficient nature of algae-mediated production of nanoparticles [15]. Phytochemical compounds such as ascorbic acid, flavonoids, citric acid, terpenes, and alkaloids are among the bioactive metabolites found in algae extracts that may function as reducing agents [16].

Kumar et al. [17] depicted that biosynthesized silver nanoparticles by *Sargassum tenerrimum* exhibited an indication of excessive anti-bacterial activity versus all the examination of pathogenic strains related to phytochemical components. Silver nanoparticles biogenically synthesized by *Sargassum myriocystum* aqueous extract exhibited antibacterial activities against different pathogen bacteria such as *Pseudomonas aeruginosa*, *Staphylococcus aureus*, *Staphylococcus epidermidis*, *Escherichia coli*, and *Proteus vulgaris* [18]. *Sargassum polycystum*-manufactured silver nanoparticles showed noteworthy activity against the pathogenic bacteria *Mycobacterium tuberculosis* [19]. Silver nanoparticles bio-fabricated using *Sargassum polycystum* exhibited antimicrobial and anticancer versus breast cancer line MCF-7 [20].

*Staphylococcus epidermidis* is a biofilm-producing commensal organism found ubiquitously on human skin and mucous membranes, as well as on animals and in the environment [21]. Its pathogenicity is mainly due to the ability to form biofilms on indwelling medical devices. In a biofilm, *S. epidermidis* is protected against attacks from the immune system and against antibiotic treatment, making *S. epidermidis* infections difficult to eradicate [22]. *Staphylococcus capitis* has been known to produce outbreaks in neonatal units across the globe, and the cause of morbidity and mortality in hospitalised infants, particularly in those with very low birth weights, is late-onset neonatal sepsis [23]. *Staphylococcus caprae* has been implicated in a variety of human infections, with the highest incidence being in bone and joint infection [24].

In this study, we focus this study on the use of the water extract of *Sargassum vulgare* an indigenous yanbu shore in Saudi Arabia in phyco-synthesis of silver nanoparticles. The bio-fabricated silver nanoparticles (Sv/Ag-NPs) were evaluated for their antioxidant, antibacterial, anticancer, and hepato-protective activities.

## 2. Result and Discussion

### 2.1. Characterization

#### 2.1.1. UV-Spectroscopy

**Figure 1:**
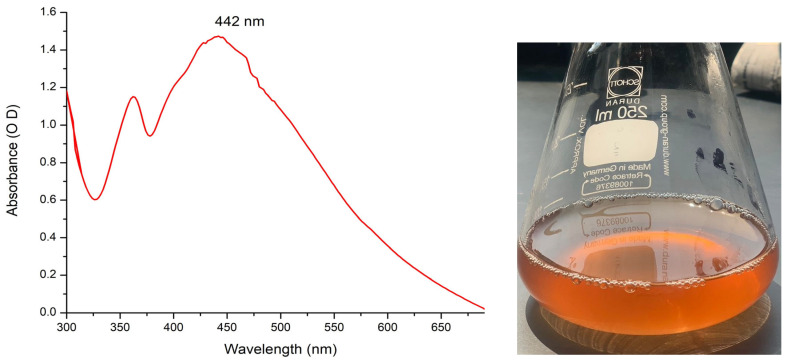
UV–visible spectrums of silver nanoparticle solutions biogenically synthesized by brown alga *S. vulgare*.

The biogenic of Sv/Ag-NPs by *S. vulgare* was confirmed by color exchange followed by measure by UV–visible spectrophotometer analysis. The UV–visible spectrophotometer of biogenic Sv/Ag-NPs shows an intense peak with strong surface plasma resonance at 422 nm with an intensity of 1.474 OD (Figure 1). Silver nanoparticles synthesized by *Sargassum tenerrimum* were definite with a UV spectral peak at 420 nm [17], synthesized by *Sargassum polycystum* at 431 nm [20], synthesized by *Sargassum cinereum* at 408 nm for silver [25]. The absorption peak of Ag-nanoparticles synthesized by *Sargassum* sp. appeared at 441 nm [26].

#### 2.1.2. Energy-Dispersive X-ray Measurements (EDX)

**Figure 2:**
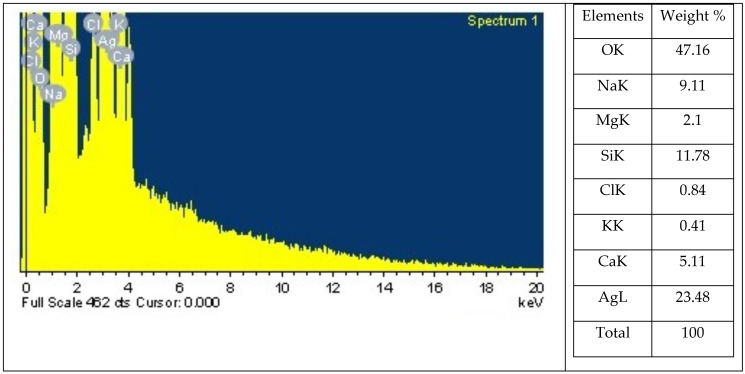
Energy dispersive X-ray spectrophotometry analysis of biogenic Sv/Ag-NPs derived from *S. vulgare*.

One analytical technique that can be used to ascertain the relative abundance of various elements in a particular sample is energy-dispersive X-ray spectroscopy. It is dependent on the interaction between a sample and an X-ray excitation source (Figure 2). The chemical elements included in a sample can be identified, and their relative abundance can be measured using EDX. The EDX analysis of Sv/Ag-NPs biogenically synthesized by *S. vulgare* has eight definite elements: O, Na, Mg, Si, Cl, K, Ca, and Ag, with percentage weights of 47.16, 9.11, 2.10, 11.78, 0.84, 0.41, 5.11, and 23.84, respectively. The EDX analysis of Ag-NPs derived from *Sargassum myriocystum* denoted the Ag ion existing in the range between 2.7 and 3 keV [18].

#### 2.1.3. X-ray Diffraction Analysis 

**Figure 3:**
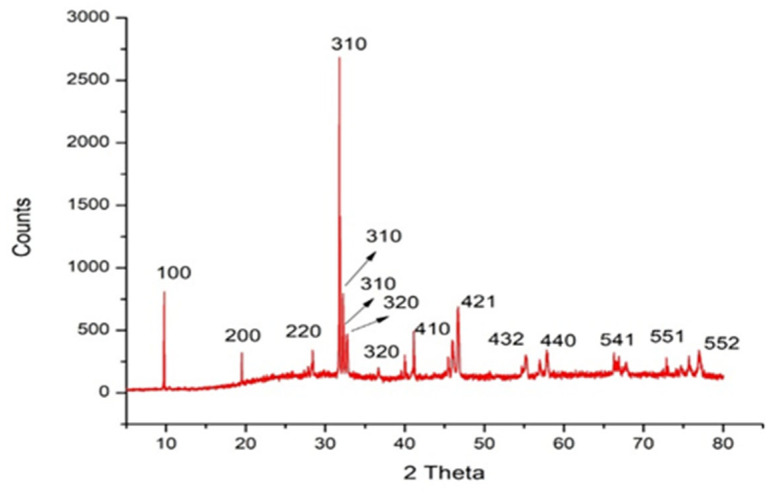
X-ray diffraction analysis of biogenic Sv/Ag-NPs derived from *S. vulgare*.

Figure 3 illustrates the X-ray diffraction (XRD) of the organized Sv/Ag-NPs. Figure 3 and Table 1 show the XRD pattern of the Sv/Ag-NPs bio-fabricated by S. vulgare, where diffraction peaks at 2θ 9.74°, 19.49, 31.76, 32.22, 32.69, 41.12, 46.65, 55.19, 57.81, 66.28, 74.72, 75.71, and 76.99 were assigned to (100), (200), (220), (310), (310), (310), (410), (421), (421), (432), (440), (541), (541), (551), (551), and (552) planes, respectively. The XRD pattern of Sv/Ag-NPs demonstrates the crystalline nature and cubic phase. Table 1 demonstrates the average size of Sv/Ag-NPs ranged between 51.8 and 79.15. The crystalline peak was attained at 2 Theta (31.766°) with an intensity of 100% and size of 76.4. All obtained peaks were sharp, confirming the crystalline patterns of Sv/Ag-NPs. The diffraction peak was quite sharp, which denoted that the silver nanoparticles are well-crystallized [27]. The green fabricated silver nanoparticles derived from *Sargassum tenerrimum* were indexed to face-centered cubic crystal constructions with four diffraction peaks of the (hkl) miller index values appointed to the planes (111), (200), (220), and (311), respectively [28].

#### 2.1.4. FT-IR Spectroscopy Analysis

**Figure 4:**
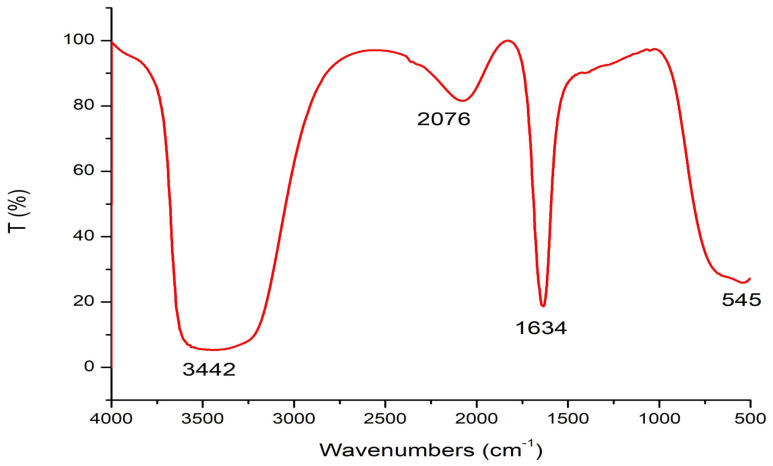
FT-IR analysis of biogenic Sv/Ag-NPs derived from *S. vulgare*.

The results in Figure 4 demonstrate the FT-IR spectroscopy analysis of Sv/Ag-NPs derived from *S. vulgare*. The outcome results show that 5 peaks were obtained from Sv/Ag-NPs. The broad peak detected at 3442 cm^−1^ matches the stretching vibration of O–H groups [29]. Peak 2076 cm^−1^ corresponded to C=N [30], while the band at 1634 cm^−1^ matched an amide [31]. These functional groups may be responsible for reducing silver into silver nanoparticles. The functional group (-OH) was the vital redox-active active group that transformed Ag+ to Ag-NPs [32]. The peak at 1640 cm^−1^ is recognized as amide I, which is responsible for the reduction of silver-to-silver nanoparticles [33].

#### 2.1.5. TEM Images

**Figure 5:**
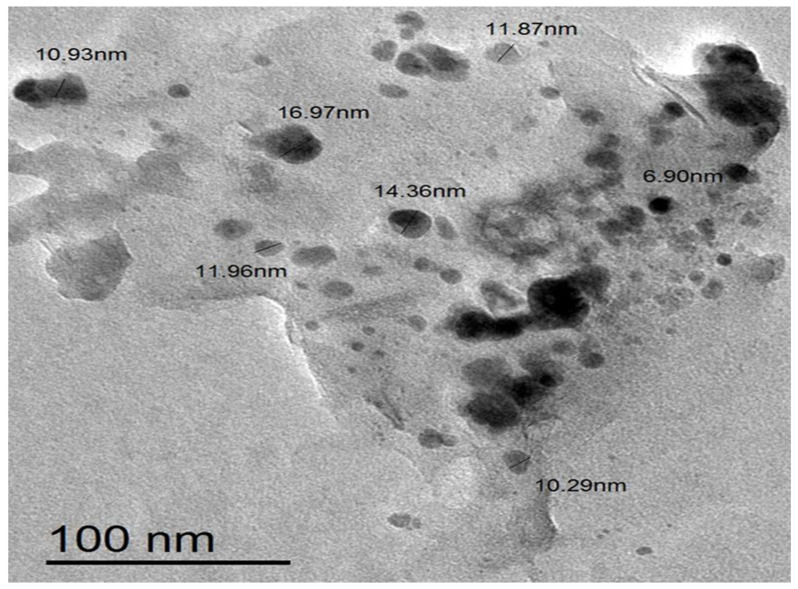
Transmission electron microscopic (TEM) image of biogenic Sv/Ag-NPs derived from *S. vulgare*.

**Figure 6:**
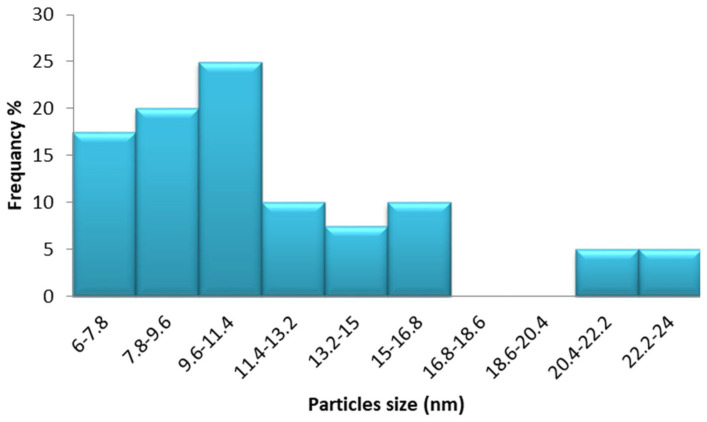
Particle size distribution of biogenic Sv/Ag-NPs derived from *S. vulgare*.

Figure 5 shows a TEM image of biosynthesized Sv/Ag-NPs derived from *S. vulgare*. The morphological studies of Sv/Ag-NPs indicated a polydispersed and spherical shape and the major range size was from 6.90 to 16.97 nm. The increased surface area produced by the fine particle size will increase the catalytic activity of the nanoparticles. Silver nanoparticles derived from *Sargassum muticum* had spherical shapes and ranged from 5 to 15 nm [34]. The results in Figure 6 validate the particle size distributions of Sv/Ag-NPs, with the major distribution range detected from 9.6 to 11.4. The diameters of silver nanoparticles bio-mediated by *Sargassum* spp. had between 2.35 nm and 11.99 nm average diameters [35].

#### 2.1.6. Zeta Potential Analysis

**Figure 7:**
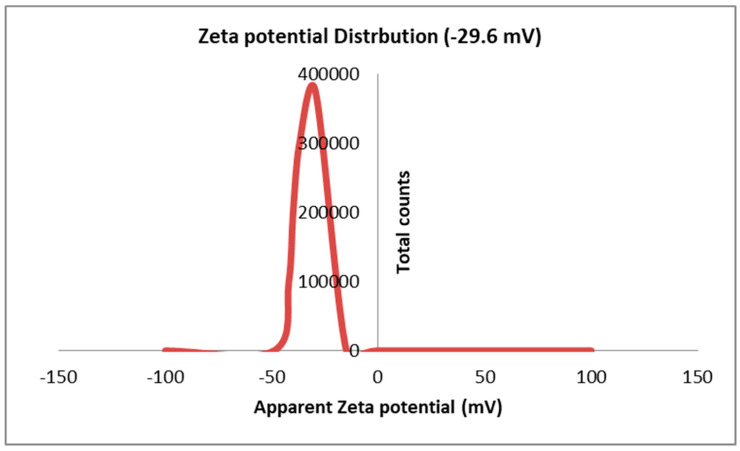
Zeta potential analysis of biogenic Sv/Ag-NPs derived from *S. vulgare*.

The results in Figure 7 display the surface charge of the Sv/Ag-NPs, which is determined by Zeta potential. The results presented in it show that the surface charge of the Sv/Ag-NPs derived from *S. vulgare* has a negative charge of −29.6 mV. The silver nanoparticles derived from *Sargassum* spp. show negative potentials, with values of less than 30 mV, which implies reliable physical stability [26]. Particles with Zeta potentials larger than ±30 mV and fewer than −30 mV are considered reflected in the stability of nanoparticles for colloidal dispersion [36]. 

### 2.2. Antioxidant Activities

**Figure 8:**
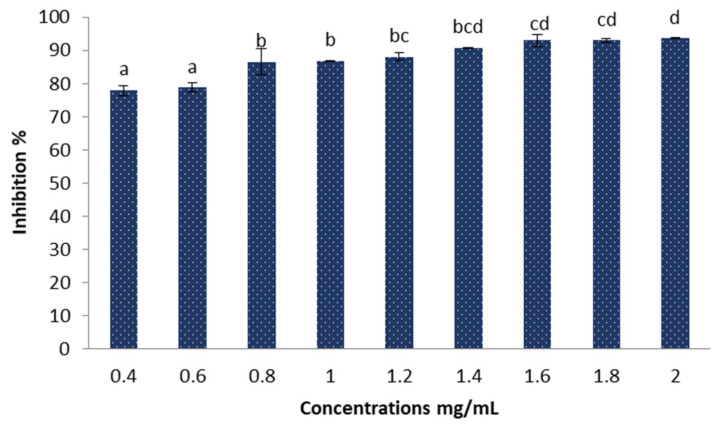
Antioxidant activities of the biogenic Sv/Ag-NPs derived from *S. vulgare* determine by DPPH. Different letters are significant values.

The results obtained in Figure 8 demonstrate the DPPH radical scavenging activity of different concentrations of Sv/Ag-NPs derived from *S. vulgare*. The antioxidant activities increase when the concentrations of Sv/Ag-NPs increase; there are no significant values between 0.4 and 0.6 mg/mL Sv/Ag-NPs, 0.8 and mg/mL Sv/Ag-NPs. The highest concentrations of Sv/Ag-NPs exhibit antioxidant activities of DPPH radical scavenging (93.63% of inhibition); meanwhile, the low concentrations exhibit 78.77% of inhibition. The results of Ag-NPs synthesized by *Sargassum wightii* have reflective reducing activity against stable free radicals [37]. The silver nanoparticles synthesized by *Sargassum polycystum* exhibit a maximum level of DPPH radical scavenging (78.2% of inhibition) [38]. Silver nanoparticles (100 µg × mL) synthesized by *Sargassum wightii* resulted in a maximum scavenging effect of 64.96% [39].

### 2.3. Anticancer Activities

**Figure 9:**
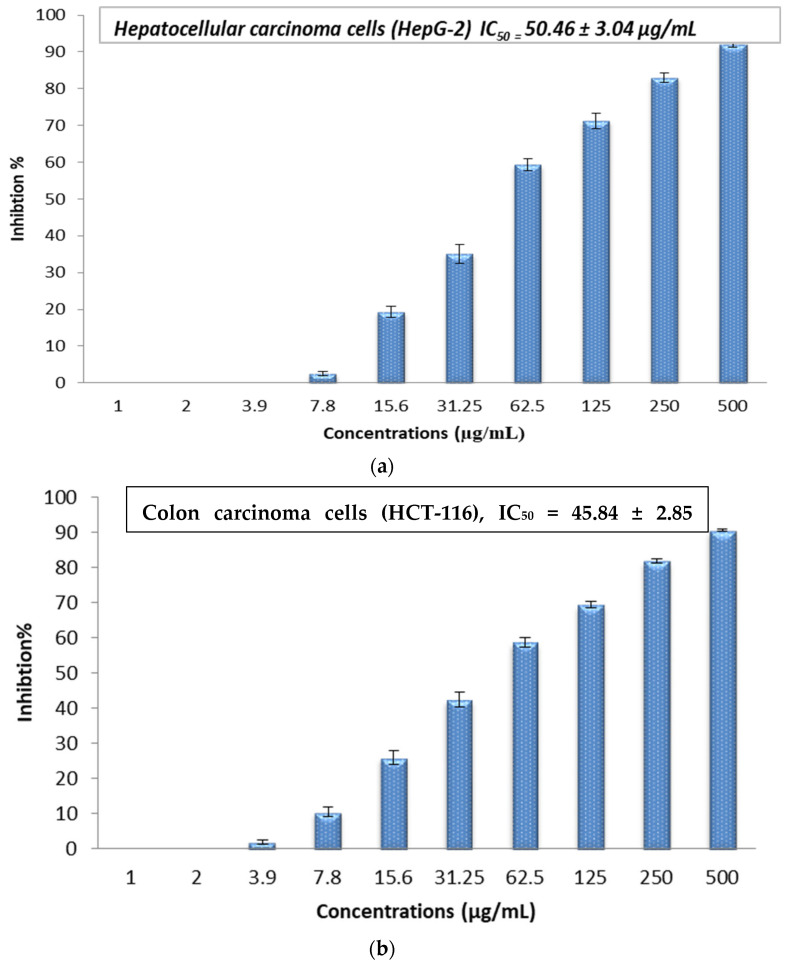

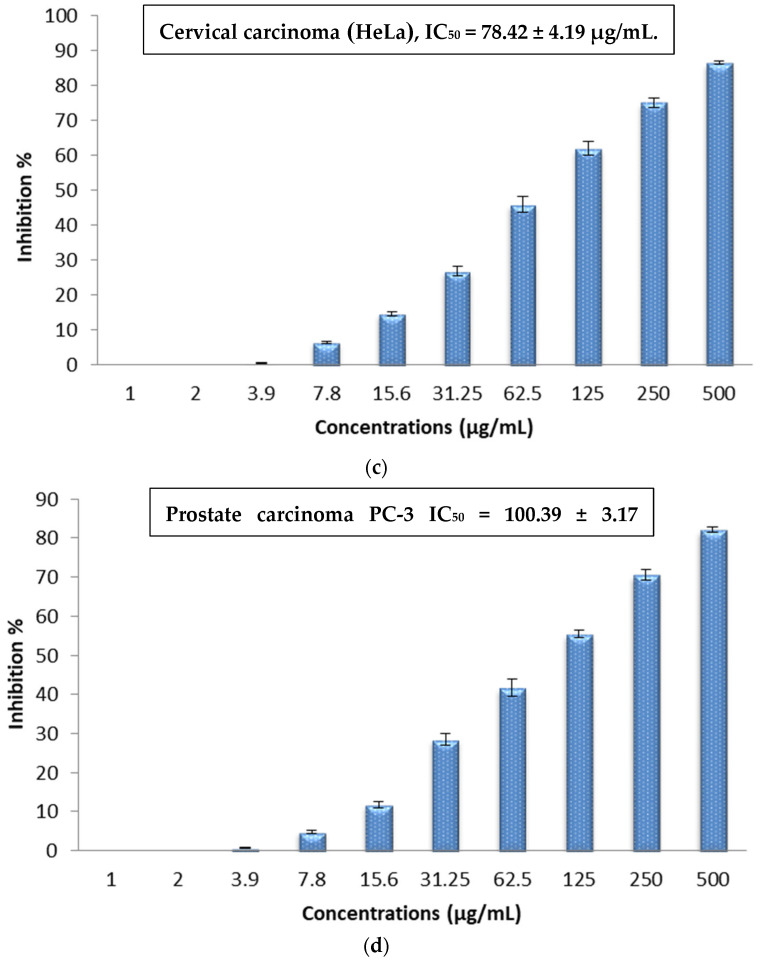
(**a**) In vitro cytotoxic activity of the biogenic Sv/Ag-NPs derived from *S. vulgare* against hepatocellular carcinoma (HepG-2). (**b**) In vitro cytotoxic activity of the biogenic Sv/Ag-NPs derived from *S. vulgare* against colon carcinoma (HCT-116). (**c**) In vitro cytotoxic activity of the biogenic Sv/Ag-NPs derived from *S. vulgare* against cervical carcinoma (HeLa). (**d**) In vitro cytotoxic activity of the biogenic Sv/Ag-NPs derived from *S. vulgare* against prostate carcinoma (PC-3).

The cytotoxic effect of the Sv/Ag-NPs derived from *S. vulgare* was evaluated in vitro against human carcinoma, such as hepatocellular carcinoma (HepG-2), colon carcinoma (HCT-116), cervical carcinoma (HeLa), and prostate carcinoma (PC-3), at different concentrations (1, 2, 3.9, 7.8, 15.6, 31.25, 62.5, 125, 250, and 500 µg/mL) (Figure 9a–d). The results demonstrate the concentration essential to produce 50% of tumor cell death (IC50); (HepG-2), (HCT-116), (HeLa), and (PC-3) was 50.46, 45.84, 78.42, and 100.39 µg/mL, respectively. The results demonstrate that cytotoxicity increases with higher concentrations of Sv/Ag-NPs against all tested cancer cells. Table 2 shows the viability of human carcinoma cells after being treated with different concentrations of biogenic Sv/Ag-NPs derived from *S. vulgare*. The results display that the Sv/Ag-NPs are the most effective against (HepG-2), (HCT-116), (HeLa), and (PC-3), respectively. The higher inhibition (92.07%) at 500 µg/mL was obtained against HepG-2. Silver nanoparticles derived from *Sargassum muticum* protected the DNA from injury, enhanced the up-regulation of tumor suppressor mRNAs, and increased antioxidant activities [40]. Silver nanoparticles decreased the viability of the HepG2 cell line in a concentration-dependent manner, and the IC50 of 75 μg/mL, due to Ag-NPs, were associated with the induction of ROS and cell apoptosis in liver hepatocellular carcinoma (HepG2) [41]. The synthesized AgNPs using chitosan possessed a cytotoxic effect against HepG2 cells, which was perceived by the analysis of the DNA ladder pattern via gel electrophoresis, and the IC50 of HepG2 cell inhibition was 48 μg/mL [42]. The AgNPs, synthesized by shrimp shell-extracted chitin as a reducing and stabilizing agent, possessed significant anticancer activity against HepG2 cells with an IC50 value of 57 ± 1.5 μg/mL; these results were confirmed by flow cytometry, which detected the apoptotic and necrotic cell death of HepG2 [43]. The silver nanoparticles bio-fabricated by *Cystoseira myrica* brown alga possessed cytotoxic activity against both MCF-7 and HepG2 [44]. The biosynthesized Ag-NPs derived from marine alga *Chaetomorpha linum* were an effective anticancer agent that could prompt apoptosis in the HCT-116 colon cells [45]. The biogenic AgNPs, derived from marine green macro-alga *Ulva lactuca*, displayed effective anticancer activity against the HCT-116 cell line due to apoptosis-mediated cell death by AgNPs [46]. The Ag-NPs, bio-fabricated by marine alga *Cladophora glomerata*, treated (HCT-116)-possessed apoptosis induction in HCT-116, as indicated by fluorescence microscopy [47]. The silver nanoparticles derived from *Hypnea musciformis* inhibited cells of colorectal cancer (HCT-116), Ehrlich ascites carcinoma (EAC), and breast cancer (MCF-7) cell line in vitro with the IC50 values of, 24.08, 40.45 and 36.95 μg/mL, respectively [48]. The Ag-NPs derived from *Sargassum myriocystum* showed effective anticancer activities against cervical HeLa cancer cells, with (IC50) noticed at 73.66 µg/mL [18]. The silver nanoparticles biogenically synthesized by *Sargassum muticum* had anticancer activity against the HeLa cancer cell line; the morphological changes of HeLa cancer cells were observed with Ag-NPs with different concentrations, which may be due to the generation of reactive oxygen species (ROS) playing a key role in the stimulation of apoptosis in all HeLa cancer cells [27]. Anticancer activities against human prostate cancer (PC-3) cells were obtained with Ag-NPS bio-fabricated by marine brown alga *Sargassum wightii*, which induces DNA fragmentation and cell death through apoptotic pathway [49]. 

### 2.4. Evaluation of In Vitro Hepatoprotective Activity

**Figure 10:**
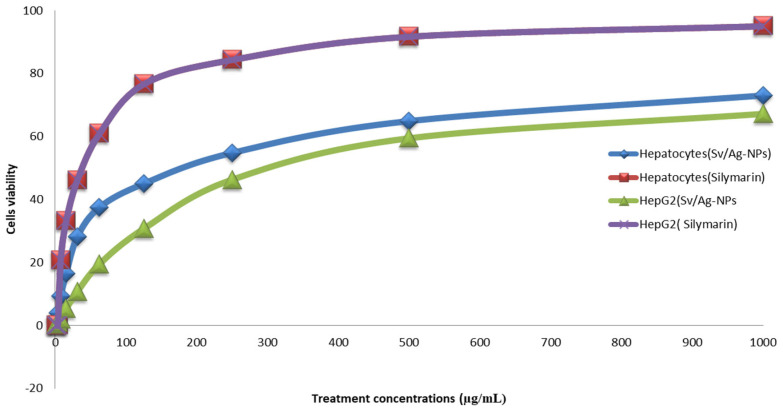
Hepatoprotective activity of biogenic Sv/Ag-NPs derived from *S. vulgare* in compared with Silymarin.

Cultured hepatocytes and Hep G2 treated with Sv/Ag-NPs and silymarin showed a significant increase in cell viability compared to the control (without treatments) Table 3 and Figure 10. There are no significant differences between the two methods which used hepatocytes and Hep G2 cells. The highest concentrations of Sv/Ag-NPs (1000 mg/mL) caused the cell viability of Hep G2 cells and hepatocytes, 67.12 and 73.05, respectively, but, in the case of silymarin, the cell viability of Hep G2 cells and hepatocytes was % 95.04, and 95.91, respectively. The lowest concentrations of Sv/Ag-NPs, which induced the cell viability of Hep G2 cells and hepatocytes, was 2 µg/mL, but, in the case of silymarin, the lowest concentrations were 7.8 and 2 µg/mL of Hep G2 cells, and hepatocytes, respectively. The results confirmed the antioxidant and hepatoprotective effect of Sv/Ag-NPs in carbon tetrachloride-induced Hep G2 cells and hepatocytes against damage by balancing or inhibiting the ROS generation at hepatotoxic conditions. Kunjiappan et al. [50] reported that the gold nanoparticles biogenically synthesized by *Azolla microphylla* extract confirmed effective antioxidant and hepatoprotective effects. The large size of silver nanoparticles, compared to the small size of silver nanoparticles, displayed more hepatoprotective capabilities [51]. Cytocompatibility screening assays support a grade of cell death produced by bionanomaterial when exposed to normal cells; the results of cytotoxicity assay revealed that silver nanoparticles, even at higher concentrations, have nontoxic normal cells (human fibroblast (L929) cell lines); these results open new possibilities for their application in various biomedical fields [52]. Strojny-Cieślak [53] reported that graphene oxide–silver and graphene oxide nanoparticles had high cytocompatibility toward human cell lines, fetal foreskin fibroblasts (HFFF2), and lung epithelial cells (A549). ZnO-Alginate/NCMs induced significant protection against Mitomycin C (MMC) [54]. Salman et al. [55] reported the oral treatment with Chitosan Nanoparticles (CNPs), and their loaded *Capparis cartilaginea* Decne extract, is safe; the chitosan nanoparticles exhibited powerful antigenotoxic properties. Silymarin can be referred to as hepatoprotective since it preserves intact liver cells or cells that have not yet sustained permanent damage by lowering oxidative stress and the ensuing cytotoxicity [56]. Silymarin has been used to inhibit NF-B, a transcription factor that regulates the expression of several genes involved in inflammation, cell defense, and cancer, to control the enzymes that exacerbate cellular damage, such as fibrosis and cirrhosis, and to increase DNA and protein synthesis [57]. The mechanisms of action of silymarin defense are the obstruction and adjustment of cell transporters, p-glycoprotein, estrogenic and nuclear receptors; silymarin has anti-inflammatory effects through TNF-α reduction, protective influences on erythrocyte lysis and cisplatin-induced acute nephrotoxicity [58].

### 2.5. Antibacterial Activities

**Figure 11:**
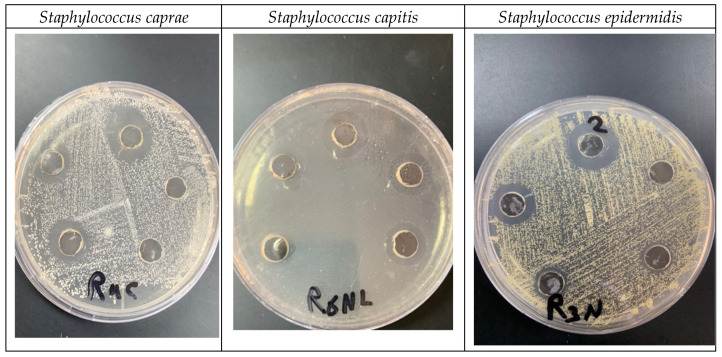
Impact of biogenic Sv/Ag-NPs derived from *S. vulgare* verses some Gram-positive bacteria.

**Figure 12:**
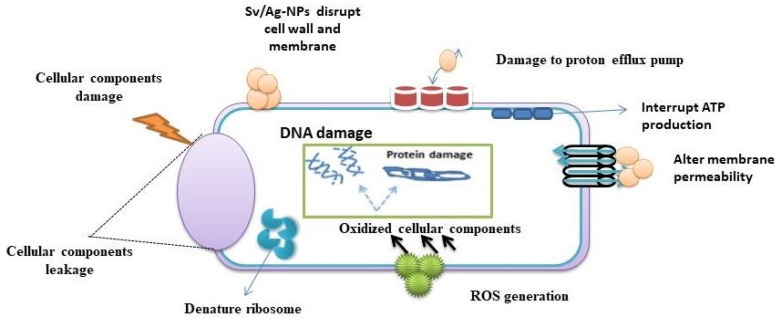
Suggested mechanism of the antibacterial actions of biogenic Sv/Ag-NPs derived from *S. vulgare*.

The results in Table 4 and Figure 11 demonstrate the impact of different concentrations (1, 0.5, 0.25, 0.125, 0.062 mg/mL) of biogenic Sv/Ag-NPs derived from *S. vulgare* verses some Gram-positive bacteria. The results demonstrate the MIC of all tested bacteria was at 0.125 mg/mL. The Sv/Ag-NPs possessed the highest activities against *Staphylococcus caprae* PP401704, *Staphylococcus capitis* PP402689, and *Staphylococcus epidermidis* PP403851, respectively. The inhibition zone composed of 1 mg/mL Sv/Ag-NPs was 21 ± 0.57 mm with *Staphylococcus caprae* PP401704, 18.33 ± 0.33 mm *Staphylococcus capitis* PP402689, and 15 ± 0.57 mm *Staphylococcus epidermidis* PP403851. Silver nanoparticles, biogenically synthesized by *Sargassum swartzii*, had antibacterial activities against *Bacillus subtilis* [59]. Biogenic Ag-NPs using *Padina* sp. were more efficient antibacterial compounds versus Gram-positive bacteria *Staphylococcus aureus* and *Bacillus subtilis* [60]. Silver nanoparticles showed higher antibacterial activity against Gram-positive bacteria *Staphylococcus capitis* than gold nanoparticles [61]. Silver nanoparticles displayed a potential antibacterial activity that was examined in vitro on *Staphylococcus epidermidis* during 24 h treatment [62]. Figure 12 displays the suggested mechanism of the antibacterial actions of biogenic Sv/Ag-NPs derived from *S. vulgare*. Silver nanoparticles adhered to the cell wall and could increase the permeability of the cytoplasmic membrane and lead to the disruption of the bacterial envelope [63]. Due to the large surface area of the nanoparticles, the nanoparticles can attach and penetrate the cell wall, making holes in the cell wall, and finally completely destroying the cell [64]. After free silver ions have entered cells, respiratory enzymes can be inhibited, generating reactive oxygen species and interrupting adenosine triphosphate production [65]. One of the main triggers for the breakdown of cell membranes and the alteration of deoxyribonucleic acid (DNA) can be reactive oxygen species. Since sulfur and phosphorus are essential parts of DNA, interactions between silver ions and these elements can disrupt DNA replication, impair cell division, or even cause microorganisms to be inhibited. Furthermore, by denaturing ribosomes in the cytoplasm, silver ions can prevent the creation of new proteins [66].

## 3. Materials and Method

### 3.1. Materials

All the chemicals used in this research were of analytical grade and applied without further purification. Chemical materials were purchased from the Saudi Chemical company. Dimethyl sulfoxide (DMSO), MTT, and trypan blue dye were purchased from Sigma (St. Louis, MO, USA). 

### 3.2. Algae Collection and Preparation

The alga *Sargassum vulgare* was collected in March 2023 from the Red Sea coast in Yanbu, Saudi Arabia, in a clean plastic bag. After the collection, the alga was washed with tap water to remove the dust suspended in it, and then it was left to dry in a ventilated area. In the laboratory, the alga dried and was ground. The alga was identified according to Taylor [67].

### 3.3. Algae Extraction

Mix one gm of alga with 100 mL of D.D. water then boil for 1 h, cool, filter by filter paper Whitman No.1, and complete to 100 mL of D.D. water [68]. 

### 3.4. Phyco-Synthesis of Nanoparticles 

Approximately 20 mL of aqueous algal extract was added drop by drop to the Erlenmeyer flask containing 90 mL (10 mM) of AgNO_3_ (0.17 gm) and heated at 60 °C till the color changed to dark brown. The initial pH of the solution was adjusted to 7.5. 

### 3.5. Characterization of Nanoparticles

The Sv/Ag-NPs solutions were characterized by using UV-visible, TEM Transmission Electron Microscope, X-ray diffraction patterns, Zeta potential, (EDS), and FT-IR, the model of all devices used in this study as in the previous study by Hamouda et al. [69].

### 3.6. Antioxidant Activity Study

The activities of Sv/Ag-NPs as free radical antioxidants were investigated by the DPPH method. One mL of several concentrations of Sv/Ag-NPs was prepared and mixed with one mL of DPPH (0.004 gm DPPH with 100 mL methanol), then left for 30 min and the absorbance was measured at 517 nm. The percentage of antioxidant activities corresponds to the following equations [70]:

$$\mathrm{Inhibition}\;\mathrm{activities}\;\%=\frac{Abs(b)-Abs(s)}{Abs(b)}\times 100$$

Abs(b) is absorbance of the blank, Abs(s) is absorbance of the sample.

### 3.7. Cytotoxicity Evaluation Using Viability Assay

Mammalian cell lines: HepG-2 cells (human hepatocellular cancer cell line), HCT-116 (human colon carcinoma), HeLa (cervical carcinoma cells), and PC-3 (prostate carcinoma cells) were attained from the American Type Culture Collection (ATCC, Rockville, MD, USA). 

The cells were cultured in RPMI-1640 media that was enhanced with 50 µg/mL of gentamycin and 10% inactivated fetal calf serum. The cells were sub-cultured two or three times each week and kept at 37 °C in a humidified environment with 5% CO_2_.

In Corning^®^ 96-well tissue culture plates, tumor cell lines were suspended in the medium at a concentration of 5 × 10^4^ cells/well to determine antitumor activities, and the plates were incubated for 24 h. After that, three duplicates of the tested compounds were put into 96-well plates, resulting in ten concentrations of each molecule. For every 96-well plate, six vehicle controls containing media or 0.5% DMSO were run as a control. The MTT test was used to assess the quantity of viable cells following a 48 h incubation period [71], and (IC50) was calculated. 

### 3.8. In Vitro Assay Hepato-Protective Effects

#### 3.8.1. Rat Hepatocyte Isolation

Hepatocyte isolation was achieved by corresponding to the collagenase perfusion methods which were pronounced by Reese and Byard [72]. Hepatocytes (1 × 10^6^ cells/mL) were located in a Krebs–Henseleit buffer (pH: 7.4) containing 12.5 mM HEPES (Sigma-Aldrich, Aberdeen, UK) and kept at 37 °C with 95% O_2_ and 5% CO_2_. 

#### 3.8.2. HepG2 Cell Line

The HepG2 cells of the human liver cell line were cultivated in DMEM (Dulbecco’s modified Eagle’s medium) including 10% fetal calf serum, penicillin (100 U), and streptomycin (100 µg).

Human liver HepG2 and Hepatocyte cells were each exposed to a medium encompassing CCl4 (1%) with/without different concentrations of the examined compounds (2, to 1000 µg/mL). Then, the viability of Hep G2 and Hepatocyte cells was estimated by MTT reduction assay. The MTT assay and the activity of dehydrogenases were used to measure the metabolic activity of live cells [71].

Hepatoprotective % = % Viability of treated group − % Viability of negative control.

EC50, the concentration required to cure 50% of intact cells, was estimated from graphic plots of the dose response curve for each concentrations.

### 3.9. Antibacterial Activities

First Muller–Hinton agar was poured into a Petri dish and solidified. Then, 10 µL of the bacterial suspension 106 cfu, (*Staphylococcus caprae* PP401704, *Staphylococcus capitis* PP402689, *Bacillus mojavensis* PP400982, and *Staphylococcus epidermidis* PP403851) was distributedover the plates using a sterilized cotton swab. Approximately 100 µL of the prepared nanoparticles Sv/Ag-NPs (1 mg/mL) were added to 0.7 mm diameter wells. Plates were incubated at 37 °C for 24 h [73].

### 3.10. Minimum Inhibitory Concentration (MIC)

The minimum inhibitory concentration (MIC) was examined using diverse concentrations of Sv/Ag-NPs 1 mg/mL to 0.065 mg/mL. Mueller–Hinton agar (MHA) was inoculated under aseptic circumstances with 10 µL of the overnight Gram-positive bacteria (*Staphylococcus caprae* PP401704, *Staphylococcus capitis* PP402689, and *Staphylococcus epidermidis* PP403851) suspension 10^6^ cfu. Wells were occupied with 100 µL serial dilutions of Sv/Ag-NPs. After 24 h of incubation at 37 °C, plates were examined, and the inhibition zone (mm) was measured.

### 3.11. Statistical Analysis

Analysis of variance (one-way ANOVA) was spent on calculating the difference among treatments and varieties via (SPSS) 16. Significant differences in resulting data were recognized at *p* < 0.05 level by using Duncan multiple ranges.

## 4. Conclusions

This study highlighted the manufacture of bio-silver nanoparticles using the aqueous extracts of marine algae *Sargassum vulgare* for its anticancer, antibacterial, and antioxidant properties, and examined the hepatoprotective effect of Hep G2 cells, and hepatocytes that were exposed to CCl4 (1%). Results indicated that the silver nanoparticles are spherical, crystalline and have negative surface charges. The silver nanoparticles exert a therapeutic potential and enhance their efficacy by reversing HepG2 and hepatocyte cells after exposure to a medium containing CCl4 (1%). Silver nanoparticles showed antioxidant, anticancer, and antibacterial activities against Gram-positive bacteria *Staphylococcus caprae* PP401704, *Staphylococcus capitis* PP402689, and *Staphylococcus epidermidis* PP403851. Green synthesis silver nanoparticles can be used in the future as therapeutic and chemo-preventive agents.

## Figures and Tables

**Table 1:** X-ray diffraction patterns of biogenic Sv/Ag-NPs derived from *S. vulgare*.

2 Theta (Å)	Crystal Size (D) nm	Intensity%	hkl
9.744	79.15	22.9	100
19.499	78.29	8.7	200
28.405	52.39	10.7	220
31.766	76.40	100	310
32.226	68.14	16.5	310
32.696	76.23	30.6	310
41.129	74.38	17.4	410
46.659	72.94	28.5	421
55.198	52.53	9.4	432
57.817	62.09	10.9	440
66.288	66.51	10.1	541
74.722	53.51	4.3	551
75.715	62.72	7.7	551
76.999	51.81	9.8	552

**Table 2:** Human carcinoma cell viability after being treated with different concentrations of biogenic Sv/Ag-NPs derived from *S. vulgare*.

Conc.; µg/mL	0	1	2	3.9	7.8	15.6	31.25	62.0	125	250	500	IC50
HepG-2	100	100	100	100	97.49	80.68	64.91	40.65	28.76	17.04	7.93	50.46
HCT-116	100	100	100	98.12	89.54	74.02	57.63	41.29	30.65	18.29	9.57	45.84
HeLa	100	100	100	99.56	93.61	85.40	73.18	54.06	38.12	24.95	13.68	78.42
PC-3	100	100	100	99.29	95.14	88.23	71.49	58.30	44.61	29.47	17.92	100.39

**Table 3:** Hepatoprotective activity of biogenic Sv/Ag-NPs derived from *S. vulgare* compared with silymarin.

Sample Conc. (µg/mL)	Sv/Ag-NPs	Silymarin	Sv/Ag-NPs	Silymarin
	(On Hep G2 Cells)		(On Rat Hepatocytes)	
1000	67.21 ± 2.37	95.04 ± 0.62	73.05 ± 0.93	95.91 ± 1.03
500	59.46 ± 2.08	91.72 ± 0.94	64.92 ± 1.46	92.43 ± 0.91
250	46.32 ± 1.94	84.33 ± 0.83	54.81 ± 0.67	87.02 ± 1.44
125	30.68 ± 1.76	76.59 ± 0.63	45.03 ± 1.42	76.54 ± 2.08
62.5	19.47 ± 0.91	60.86 ± 1.44	37.49 ± 1.57	57.28 ± 1.96
31.25	10.68 ± 0.74	46.01 ± 1.09	28.12 ± 1.43	44.73 ± 1.29
15.6	5.31 ± 0.63	33.05 ± 1.65	16.45 ± 0.71	32.56 ± 1.42
7.8	2.07 ± 0.51	20.58 ± 0.93	9.31 ± 0.65	24.82 ± 0.74
3.9	0.98 ± 0.34	0	4.06 ± 0.28	16.59 ± 0.67
2	0.32 ± 0.16	0	1.87 ± 0.49	11.23 ± 0.25
0	0	0	0	0
IC50 (µg/mL)	320	39.64	188.52	44.37

**Table 4:** Minimum inhibitory concentration (MIC) assays of biogenic Sv/Ag-NPs derived from *S. vulgare* verses some Gram-positive bacteria (determined by inhibition zone).

Concentrations mg/mL	1	0.5	0.25	0.125	0.062
*Staphylococcus caprae* PP401704	21 ± 0.57	17.66 ± 0.33	17.33 ± 0.33	14.66 ± 0.33	0
*Staphylococcus capitis* PP402689	18.33 ± 0.33	17.33 ± 0.33	16 ± 0.0	14.33 ± 0.33	0
*Staphylococcus epidermidis* PP403851	15 ± 0.57	13 ± 0.57	11.66 ± 0.33	8.33 ± 0.330	0

## Data Availability

Data will be made available on request.

## References

[1] Lewandowska A., Rudzki G., Lewandowski T., Rudzki S. (2021). The problems and needs of patients diagnosed with cancer and their caregivers. Int. J. Environ. Res. Public Health.

[2] Asrani S.K., Devarbhavi H., Eaton J., Kamath P.S. (2019). Burden of liver diseases in the world. J. Hepatol..

[3] Abou Seif H.S. (2016). Physiological changes due to hepatotoxicity and the protective role of some medicinal plants. Beni-Suef Univ. J. Basic Appl. Sci..

[4] Doron S., Gorbach S.L. (2008). Bacterial infections: Overview. International Encyclopedia of Public Health.

[5] Rather M.A., Gupta K., Bardhan P., Borah M., Sarkar A., Eldiehy K.S., Mandal M. (2021). Microbial biofilm: A matter of grave concern for human health and food industry. J. Basic Microbiol..

[6] Baldevraj R.M., Jagadish R.S. (2011). Incorporation of chemical antimicrobial agents into polymeric films for food packaging. Multifunctional and Nanoreinforced Polymers for Food Packaging.

[7] Wang L., Hu C., Shao L. (2017). The antimicrobial activity of nanoparticles: Present situation and prospects for the future. Int. J. Nanomed..

[8] Mustafa H.N., El Awdan S.A., Hegazy G.A. (2013). Protective role of antioxidants on thioacetamide-induced acute hepatic encephalopathy: Biochemical and ultrastructural study. Tissue Cell.

[9] Pandit C., Roy A., Ghotekar S., Khusro A., Islam M.N., Emran T.B., Lam S.E., Khandaker M.U., Bradley D.A. (2022). Biological agents for synthesis of nanoparticles and their applications. J. King Saud Univ.-Sci..

[10] Elzoheiry A., Ayad E., Omar N., Elbakry K., Hyder A. (2022). Anti-liver fibrosis activity of curcumin/chitosan-coated green silver nanoparticles. Sci. Rep..

[11] Lei Z., Karim A. (2021). The challenges and applications of nanotechnology against bacterial resistance. J. Vet. Pharmacol. Ther..

[12] Gu L., Zhang F., Wu J., Zhuge Y. (2022). Nanotechnology in drug delivery for liver fibrosis. Front. Mol. Biosci..

[13] Vargas-Mendoza N., Madrigal-Santillán E., Morales-González Á., Esquivel-Soto J., Esquivel-Chirino C., González-Rubio M.G.L.Y., Gayosso-de-Lucio J.A., Morales-González J.A. (2014). Hepatoprotective effect of silymarin. World J. Hepatol..

[14] Wellington K., Jarvis B. (2001). Silymarin: A review of its clinical properties in the management of hepatic disorders. BioDrugs.

[15] Gonçalves A., Fernandes M., Lima M., Gomes J.P., Silva F., Castro S., Gomes A.C. (2023). Nanotechnology to the Rescue: Therapeutic Strategies Based on Brown Algae for Neurodegenerative Diseases. Appl. Sci..

[16] Santhoshkumar J., Rajeshkumar S., Kumar S.V. (2017). Phyto-assisted synthesis, characterization and applications of gold nanoparticles: A review. Biochem. Biophys. Rep..

[17] Kumar P., Senthamil Selvi S., Lakshmi Prabha A., Prem Kumar K., Ganeshkumar R.S., Govindaraju M. (2012). Synthesis of silver nanoparticles from *Sargassum tenerrimum* and screening phytochemicals for its antibacterial activity. Nano Biomed. Eng..

[18] Balaraman P., Balasubramanian B., Kaliannan D., Durai M., Kamyab H., Park S., Maruthupandian A. (2020). Phyco-synthesis of silver nanoparticles mediated from marine algae *Sargassum myriocystum* and its potential biological and environmental applications. Waste Biomass Valorization.

[19] Thiurunavukkarau R., Shanmugam S., Subramanian K., Pandi P., Muralitharan G., Arokiarajan M., Kasinathan K., Sivaraj A., Kalyanasundaram R., AlOmar S.Y. (2022). Silver nanoparticles synthesized from the seaweed *Sargassum polycystum* and screening for their biological potential. Sci. Rep..

[20] Thangaraju N., Venkatalakshmi R.P., Chinnasamy A., Kannaiyan P.J.A.N.B.E. (2012). Synthesis of silver nanoparticles and the antibacterial and anticancer activities of the crude extract of *Sargassum polycystum* C. Agardh. Nano Biomed. Eng..

[21] Kleinschmidt S., Huygens F., Faoagali J., Rathnayake I.U., Hafner L.M. (2015). Staphylococcus epidermidis as a cause of bacteremia. Future Microbiol..

[22] Vuong C., Otto M. (2002). *Staphylococcus epidermidis* infections. Microbes Infect..

[23] Heath V., Cloutman-Green E., Watkin S., Karlikowska M., Ready D., Hatcher J., Pearce-Smith N., Brown C., Demirjian A. (2023). *Staphylococcus capitis*: Review of Its Role in Infections and Outbreaks. Antibiotics.

[24] Gowda A., Pensiero A.L., Packer C.D., Pensiero A. (2018). *Staphylococcus caprae*: A skin commensal with pathogenic potential. Cureus.

[25] Mohandass C., Vijayaraj A.S., Rajasabapathy R., Satheeshbabu S., Rao S.V., Shiva C., De-Mello I. (2013). Biosynthesis of silver nanoparticles from marine seaweed *Sargassum cinereum* and their antibacterial activity. Indian J. Pharm. Sci..

[26] López-Miranda J.L., Esparza R., González-Reyna M.A., España-Sánchez B.L., HernandezMartinez A.R., Silva R., Estévez M. (2021). Sargassum Influx on the Mexican Coast: A Source for Synthesizing Silver Nanoparticles with Catalytic and Antibacterial Properties. Appl. Sci..

[27] Fan L., Zhang H., Gao M., Zhang M., Liu P., Liu X. (2020). Cellulose nanocrystals/silver nanoparticles: In-situ preparation and application in PVA films. Holzforschung.

[28] Veeragoni D., Deshpande S.S., Singh V., Misra S., Mutheneni S.R. (2023). In vitro and in vivo antimalarial activity of green synthesized silver nanoparticles using *Sargassum tenerrimum*—A marine seaweed. Acta Trop..

[29] Indana M.K., Gangapuram B.R., Dadigala R., Bandi R., Guttena V. (2016). A novel green synthesis and characterization of silver nanoparticles using gum tragacanth and evaluation of their potential catalytic reduction activities with methylene blue and Congo red dyes. J. Anal. Sci. Technol..

[30] Kamalakannan S., Gobinath C., Ananth S. (2014). Synthesis and characterization of fungus mediated silver nanoparticle for toxicity on filarial vector, *Culex quinquefasciatus*. Int. J. Pharm. Sci. Rev. Res..

[31] Vankar P.S., Shukla D. (2012). Biosynthesis of silver nanoparticles using lemon leaves extract and its application for antimicrobial finish on fabric. Appl. Nanosci..

[32] Peng H., Guo H., Gao P., Zhou Y., Pan B., Xing B. (2021). Reduction of silver ions to silver nanoparticles by biomass and biochar: Mechanisms and critical factors. Sci. Total Environ..

[33] McIntyre T.C., McCutcheon S.C., Schnoor J.L. (2003). Phytoremediation: Transformation and Control of Contaminants.

[34] Azizi S., Namvar F., Mahdavi M., Ahmad M.B., Mohamad R. (2013). Biosynthesis of silver nanoparticles using brown marine macroalga, *Sargassum muticum* aqueous extract. Materials.

[35] Vinayagam R., Nagendran V., Goveas L.C., Narasimhan M.K., Varadavenkatesan T., Chandrasekar N., Selvaraj R. (2023). Structural characterization of marine macroalgae derived silver nanoparticles and their colorimetric sensing of hydrogen peroxide. Mater. Chem. Phys..

[36] Gurunathan S. (2015). Biologically synthesized silver nanoparticles enhances antibiotic activity against Gram-negative bacteria. J. Ind. Eng. Chem..

[37] Ponmani J., Kanakarajan S., Selvaraj R., Kamalanathan A. (2020). Antioxidant properties of green synthesized silver nanoparticles from *Sargassum wightii*. Saudi J. Med. Pharm. Sci..

[38] Palanisamy S., Rajasekar P., Vijayaprasath G., Ravi G., Manikandan R., Prabhu N.M. (2017). A green route to synthesis silver nanoparticles using *Sargassum polycystum* and its antioxidant and cytotoxic effects: An in vitro analysis. Mater. Lett..

[39] Deepak P., Amutha V., Birundha R., Sowmiya R., Kamaraj C., Balasubramanian V., Balasubramani G., Aiswarya D., Arul D., Perumal P. (2018). Facile green synthesis of nanoparticles from brown seaweed *Sargassum wightii* and its biological application potential. Adv. Nat. Sci. Nanosci. Nanotechnol..

[40] Alshehri M.A. (2019). Hepatoprotective impact of seaweed (*Sargassum muticum*) nanoparticles against diethylnitrosamine promoted progression of liver tumor in male rats. J. Biochem. Technol..

[41] Ahmadian E., Dizaj S.M., Rahimpour E., Hasanzadeh A., Eftekhari A., Halajzadeh J., Ahmadian H. (2018). Effect of silver nanoparticles in the induction of apoptosis on human hepatocellular carcinoma (HepG2) cell line. Mater. Sci. Eng. C.

[42] Priya K., Vijayakumar M., Janani B. (2020). Chitosan-mediated synthesis of biogenic silver nanoparticles (AgNPs), nanoparticle characterisation and in vitro assessment of anticancer activity in human hepatocellular carcinoma HepG2 cells. Int. J. Biol. Macromol..

[43] Vijayakumar M., Priya K., Ilavenil S., Janani B., Vedarethinam V., Ramesh T., Arasu M.V., Al-Dhabi N.A., Kim Y.-O., Kim H.J. (2020). Shrimp shells extracted chitin in silver nanoparticle synthesis: Expanding its prophecy towards anticancer activity in human hepatocellular carcinoma HepG2 cells. Int. J. Biol. Macromol..

[44] Mohamed R.M., Fawzy E.M., Shehab R.A., Abdel-Salam M.O., Salah El Din R.A., Abd El Fatah H.M. (2022). Production, characterization, and cytotoxicity effects of silver nanoparticles from Brown alga (*Cystoseira myrica*). J. Nanotechnol..

[45] Acharya D., Satapathy S., Somu P., Parida U.K., Mishra G. (2021). Apoptotic effect and anticancer activity of biosynthesized silver nanoparticles from marine algae *Chaetomorpha linum* extract against human colon cancer cell HCT-116. Biol. Trace Elem. Res..

[46] Acharya D., Satapathy S., Yadav K.K., Somu P., Mishra G. (2022). Systemic evaluation of mechanism of cytotoxicity in human colon cancer HCT-116 cells of silver nanoparticles synthesized using marine algae Ulva lactuca extract. J. Inorg. Organomet. Polym. Mater..

[47] Acharya D., Satapathy S., Thathapudi J.J., Somu P., Mishra G. (2022). Biogenic synthesis of silver nanoparticles using marine algae *Cladophora glomerata* and evaluation of apoptotic effects in human colon cancer cells. Mater. Technol..

[48] Ghose R., Asaduzzaman A.K.M., Hasan I., Kabir S.R. (2022). *Hypnea musciformis*-mediated Ag/AgCl-NPs inhibit pathogenic bacteria, HCT-116 and MCF-7 cells’ growth in vitro and Ehrlich ascites carcinoma cells in vivo in mice. IET Nanobiotechnol..

[49] Ponmani J., Kanakarajan S., Selvaraj R., Kamalanathan A. (2021). Induced Apoptotic Potential of Green Synthesized AgNPs from *Sargassum wightii* on Human Prostate Cancer (PC-3) Cells. Chettinad Health City Med. J..

[50] Kunjiappan S., Bhattacharjee C., Chowdhury R. (2015). In vitro antioxidant and hepatoprotective potential of *Azolla microphylla* phytochemically synthesized gold nanoparticles on acetaminophen–induced hepatocyte damage in *Cyprinus carpio* L.. Vitr. Cell. Dev. Biol.-Anim..

[51] Elfaky M.A., Sirwi A., Ismail S.H., Awad H.H., Gad S.S. (2022). Hepatoprotective effect of silver nanoparticles at two different particle sizes: Comparative study with and without Silymarin. Curr. Issues Mol. Biol..

[52] Jadhav K., Deore S., Dhamecha D., Hr R., Jagwani S., Jalalpure S., Bohara R. (2018). Phytosynthesis of silver nanoparticles: Characterization, biocompatibility studies, and anticancer activity. ACS Biomater. Sci. Eng..

[53] Strojny-Cieślak B., Jaworski S., Wierzbicki M., Pruchniewski M., Sosnowska-Ławnicka M., Szczepaniak J., Chwalibóg E.S. (2023). The cytocompatibility of graphene oxide as a platform to enhance the effectiveness and safety of silver nanoparticles through in vitro studies. Environ. Sci. Pollut. Res..

[54] Hamouda R.A., Salman A.S., Alharbi A.A., Alhasani R.H., Elshamy M.M. (2021). Assessment of the Antigenotoxic Effects of Alginate and ZnO/Alginate–Nanocomposites Extracted from Brown Alga *Fucus vesiculosus* in Mice. Polymers.

[55] Salman A.S., Alkhatib S.N., Ahmed F.M., Hamouda R.A. (2023). Chitosan Nanoparticles Loaded with *Capparis cartilaginea* Decne Extract: Insights into Characterization and Antigenotoxicity In Vivo. Pharmaceutics.

[56] Mohammed H.A., Khan R.A. (2022). Anthocyanins: Traditional Uses, Structural and Functional Variations, Approaches to Increase Yields and Products’ Quality, Hepatoprotection, Liver Longevity, and Commercial Products. Int. J. Mol. Sci..

[57] Gillessen A., Schmidt H.H.-J. (2020). Silymarin as Supportive Treatment in Liver Diseases: A Narrative Review. Adv. Ther..

[58] Karimi G., Vahabzadeh M., Lari P., Rashedinia M., Moshiri M. (2011). “Silymarin”, a promising pharmacological agent for treatment of diseases. Iran. J. Basic Med. Sci..

[59] Solanki A.D., Patel I. (2022). *Sargassum swartzii*: A source of silver nanoparticles, synthesis and its antibacterial activity. Egypt. J. Agric. Res..

[60] Bhuyar P., Rahim M.H.A., Sundararaju S., Ramaraj R., Maniam G.P., Govindan N. (2020). Synthesis of silver nanoparticles using marine macroalgae *Padina* sp. and its antibacterial activity towards pathogenic bacteria. Beni-Suef Univ. J. Basic Appl. Sci..

[61] Amin R.M., Mohamed M.B., Ramadan M.A., Verwanger T., Krammer B. (2009). Rapid and sensitive microplate assay for screening the effect of silver and gold nanoparticles on bacteria. Nanomedicine.

[62] Kalishwaralal K., BarathManiKanth S., Pandian S.R.K., Deepak V., Gurunathan S. (2010). Silver nanoparticles impede the biofilm formation by *Pseudomonas aeruginosa* and *Staphylococcus epidermidis*. Colloids Surf. B Biointerfaces.

[63] Khorrami S., Zarrabi A., Khaleghi M., Danaei M., Mozafari M.R. (2018). Selective cytotoxicity of green synthesized silver nanoparticles against the MCF-7 tumor cell line and their enhanced antioxidant and antimicrobial properties. Int. J. Nanomed..

[64] Sindi H.A., Hamouda R.A., Alhazmi N.M., Abdel-Hamid M.S. (2024). Functionalized gold nanoparticles coated with bacterial alginate and their antibacterial and anticancer activities. Green Process. Synth..

[65] Ramkumar V.S., Pugazhendhi A., Gopalakrishnan K., Sivagurunathan P., Saratale G.D., Dung T.N.B., Kannapiran E. (2017). Biofabrication and characterization of silver nanoparticles using aqueous extract of seaweed *Enteromorpha compressa* and its biomedical properties. Biotechnol. Rep..

[66] Yin I.X., Zhang J., Zhao I.S., Mei M.L., Li Q., Chu C.H. (2020). The antibacterial mechanism of silver nanoparticles and its application in dentistry. Int. J. Nanomed..

[67] Taylor W.R. (1985). Marine Algae of the Eastern Tropical and Subtropical Coasts of the America.

[68] Hamouda R.A., Alharthi M.A., Alotaibi A.S., Alenzi A.M., Albalawi D.A., Makharita R.R. (2023). Biogenic Nanoparticles Silver and Copper and Their Composites Derived from Marine Alga *Ulva lactuca*: Insight into the Characterizations, Antibacterial Activity, and Anti-Biofilm Formation. Molecules.

[69] Hamouda R.A., Alharbi A.A., Al-Tuwaijri M.M., Makharita R.R. (2023). The Antibacterial Activities and Characterizations of Biosynthesized Zinc Oxide Nanoparticles, and Their Coated with Alginate Derived from *Fucus vesiculosus*. Polymers.

[70] Sarikurkcu C., Arisoy K., Tepe B., Cakir A., Abali G., Mete E. (2009). Studies on the antioxidant activity of essential oil and different solvent extracts of *Vitex agnus castus* L. fruits from Turkey. Food Chem. Toxicol..

[71] Mosmann T. (1983). Rapid colorimetric assay for cellular growth and survival: Application to proliferation and cytotoxicity assays. J. Immunol. Methods.

[72] Reese J.A., Byard J.L. (1981). Isolation and culture of adult hepatocytes from liver biopsies. In Vitro.

[73] Hamouda R.A., Makharita R.R., Qarabai F.A.K., Shahabuddin F.S., Saddiq A.A., Bahammam L.A., El-Far S.W., Bukhari M.A., Elaidarous M.A., Abdella A. (2024). Antibacterial Activities of Ag/Cellulose Nanocomposites Derived from Marine Environment Algae against Bacterial Tooth Decay. Microorganisms.

